# Attention speed and anterior cingulate cortex volume in female and male veterans with suicide ideation and attempts

**DOI:** 10.3389/fpsyt.2024.1495046

**Published:** 2025-01-22

**Authors:** Erin C. McGlade, James R. Yancey, Keenan E. Roberts, Audrey Elias, Chelsea Carson, Jiyoung Ma, Margaret R. Legarreta, Deborah A. Yurgelun-Todd

**Affiliations:** ^1^ Diagnostic Neuroimaging Lab, University of Utah, Salt Lake City, UT, United States; ^2^ Department of Psychiatry, Huntsman Mental Health Institute, University of Utah, Salt Lake City, UT, United States; ^3^ Department of Veterans Affairs, Rocky Mountain MIRECC, Salt Lake City, UT, United States; ^4^ Department of Veterans Affairs, Sheridan VA Health Care System, Sheridan, WY, United States

**Keywords:** suicide, attention, anterior cingulate cortex, magnetic resonance imaging, veterans

## Abstract

**Introduction:**

An average of 17.5 Veterans died by suicide each day in 2021, highlighting the importance of research and prevention efforts aimed at suicide risk. Attentional processes have emerged as a possible predictor of suicide behaviors (SB), yet associated neural correlates remain understudied, particularly in the Veteran population.

**Methods:**

The current study examines sustained and selective attention performance as indexed by the Ruff 2 & 7 Selective Attention Test and anterior cingulate cortex (ACC) volume as they relate to SB in Veterans. A subset of Veterans also completed a structural magnetic resonance imaging protocol. Participants were grouped on history of suicidal ideation (SI), suicide attempt (SA), and no SB (HC).

**Results:**

Analyses from the Ruff 2 & 7 test showed that Veterans with a history of SA performed more slowly on Automatic Detection Speed (ADS) and Controlled Search Speed (CSS) compared to Veterans with SI and no SB. SI and SA group differences on ADS and CSS remained after Bonferroni correction, and CSS differences remained after controlling for depressive and anxious symptoms. There were no between-group differences on Ruff 2 & 7 Accuracy measures. When analyses were divided by sex, males with a history of SA performed more slowly than SI and HC on ADS and more slowly on CSS and Total Speed than males with a history of SI. Results remained significant after controlling for depressive and anxious symptoms. When Bonferroni corrections were applied, males with a history of SA performed more slowly on ADS, CSS, and Total Speed compared to males with a history of SI. Female Veterans with a history of SA performed more slowly than female HC on CSS only; however, these findings were no longer significant after controlling for depressive and anxious symptoms. No significant differences were found between female groups on ADS or Total Speed. Measures of left rostral ACC gray matter (GM) volume for the combined female and male Veteran sample were positively correlated with ADS and CSS scores in HC but not SA. Conversely, right rostral ACC GM volume negatively correlated with ADS and CSS scores in the SA group but not HC. Right rostral ACC white matter volume correlated positively with ADS and CSS in HC.

**Discussion:**

These findings highlight associations between attention speed, ACC volume, and SB even after controlling for acute mood symptoms, in addition to emphasizing the importance of including sex in analyses.

## 1 Introduction

The Office of Mental Health and Suicide Prevention reports that 6,392 Veterans died by suicide in 2021, which is an average of 17.5 Veteran suicide deaths per day (1). These rates highlight the importance of research focused on suicide, and the Department of Veterans Affairs continues to prioritize suicide risk and prevention in Veterans. While it is recognized that suicide behavior (SB), including suicide ideation (SI) and suicide attempts (SA), is the result of a complicated number of interacting factors, one important focus has been the association between neurocognitive performance and SB (2).

A 2019 review of studies on suicide and cognition highlights early methods of qualitatively examining suicide notes of those who died by suicide, which showed cognitive rigidity or decreased problem solving (3). Researchers have since identified a number of other neurocognitive correlates to SB, including deficits in cognitive flexibility, executive function, memory, and attention (3–5). Studies examining neurocognitive performance in suicide attempters specifically have identified executive function, selective attention, inhibition, working memory, and decision making as the most common areas of poor performance (6).

A key part of cognition, attention, is not a unitary phenomenon but a complex function comprised of multiple informational processing abilities (7). Sustained attention refers to the ability to maintain attention on a stimulus while selective attention refers to the ability to maintain attention on the goal target while ignoring irrelevant targets (8). Reaction time is defined as the time it takes an individual to respond to the stimuli. Attentional tasks may also be divided according to the form of stimuli, such as visual and auditory tasks. Visual attentional demands are evident in cancellation tasks where a person crosses out numbers, and auditory attentional tasks include those requiring repeating numbers out loud. Attention also has been identified as a core function for other neuropsychological processes including memory and cognitive productivity such that even minor deficits in attention functions can affect other cognitive processes (9). For instance, if an individual has poor attention, they will not be able to attend, encode, or then recall information because of the initial impaired attention.

Due to the complexity of attention, research relating it to SB has used a variety of assessment measures, including the Tests of Variables of Attention (TOVA), in which the person is asked to push a button in response to stimuli on a computer screen (10) and Comprehensive Attention Test (CAT), in which a person is asked to respond to shapes other than X (11). Research on attention and SB has found deficits in reaction times and psychomotor performance in depressed individuals with and without a history of SB (12). Individuals with depression also have shown poorer attentional functioning measured by a modified Stroop, particularly in individuals with a history of SA (13). Interestingly, some deficits in attention have been below self-report thresholds, as adolescents with SB have shown impaired attention on the TOVA behavioral task while not self-reporting impaired attention (10). In that study depressive symptoms did not correlate with impaired attention performance. The association between attention and SB also has been shown in international samples with Kim and colleagues reporting an association between omission and commission errors with SI on the CAT. Findings remained after controlling for depressed mood (11). Interactions with gender also were noted, as females in the high omission error group showed higher SI scores.

The prefrontal cortex has been shown to play a critical role in carrying out attentional functions (7, 14). The anterior cingulate cortex (ACC) has been considered a key cortical area during the processing of cognitively demanding information, as it has been shown to be associated with a number of functions including response selection, inhibition, and attention (15). The ACC also has been identified as important in sustained attention in both animal models and humans (16). Studies using the Flanker test and Stroop have shown that the ACC is involved in monitoring conflict, while other frontal regions respond to the conflict (17). Attention shifting and dual task-performance also have been related to brain activity in the ACC of healthy controls (18). ACC volume and functional activation differences have been shown in healthy adolescents and adults compared to those with Attention Deficit Hyperactivity Disorder (ADHD) (19). Together these studies highlight the role of the ACC in attentional performance.

Alterations of the ACC also have been suggested as potential biomarkers for SB. A recent review of magnetic resonance imaging (MRI) studies found reduced volume of the ACC in individuals with SB and those with non-suicidal self-injury (NSSI) (20). Huber and colleagues extended these findings to Veterans, showing reduced WM volume in the left rostral ACC of Veterans with a history of SA when compared to Veterans with a history of SI (21). A meta-analysis of patients with a history of SB found an increase in ACC activation during tasks with emotional stimuli and lower activation during cognitive tasks (22). More recently Ambrosi and colleagues examined resting state functional connectivity (rsFC) in psychiatric inpatients with SB and major depressive or bipolar disorder, inpatients without a history of SB, and HC (23). They found that individuals with a history of SB showed altered rsFC of the ACC. The rostral ACC emerged as a region of particular importance, as participants in the SB+ group showed higher rsFC between the left rostral and right dorsal ACC with the visual cortex in addition to decreased rsFC between the left rostral and right dorsal ACC and cingulate and frontal clusters. Importantly, connectivity between the left rostral ACC and Brodmann’s area 18 (occipital lobe) distinguished SB+ from SB- patients with 75% accuracy. These findings together support the importance of considering the rostral ACC in studies of the neurobiology of suicide.

In summary, prior research has shown associations between attention and SB; attention and ACC; and suicide and ACC. However, to our knowledge no prior studies have looked at attentional performance differently by history of SB related to ACC. The present study therefore examined sustained and selective attention performance and ACC volume by history of SB. We hypothesized that individuals with SB would show decreased attention compared to HC and that ACC volume would be related to attentional performance differently in those with a history of SA, compared to those with SI and HC. Furthermore, we completed exploratory analyses based on sex given prior findings on sex differences in SB (1, 24, 25).

## 2 Materials and methods

### 2.1 Participants

One hundred twenty-nine participants were recruited for study inclusion with 77 participants completing MRI. Participants were recruited from the George E. Wahlen Health Care System and surrounding areas via fliers and word of mouth. The Institutional Review board (IRB) for the University of Utah and VASLC approved all study procedures including informed consent. To be eligible for the study, participants had to be Veterans between ages 18 and 55. Measures of interest included MRI and cognitive performance; therefore, individuals were excluded if they had metal implanted within the body; pregnancy; claustrophobia; history of electroconvulsive therapy (ECT); significant medical or neurological illness that might affect cognitive function; and estimated IQ less than 80 to ensure proper consent and average overall cognitive functioning.

### 2.2 Attention measures

One hundred twenty-nine Veteran participants completed the Ruff 2 & 7 Selective Attention Test (Ruff 2 & 7), a visual search and cancellation task in which participants are asked to cross out all 2’s and 7’s that are embedded in a series of other numbers (Controlled Search) or capital letters (Automatic Detection) (8). This test yields scores for Automatic Detection Speed (ADS) and Controlled Search Speed (CSS) in addition to Automatic Detection Accuracy (ADA) and Controlled Search Accuracy (CSA). Speed scores reflect how many targets the participant crosses out in the given time and accuracy scores reflect the number of targets correctly identified as compared to the number of errors. Automatic Detection scores are thought to reflect more automatic target selection, whereas Controlled Search scores are thought to be more effortful, as the participant must identify the target numbers embedded amongst other numbers (8). The Total Speed score is the total number of ADS and CSS targets crossed out in the given time.

### 2.3 Mood and suicide behavior measures

Veterans also completed a structured interview assessing history of suicide ideation and attempts (Columbia Suicide Severity Rating Scales (CSSRS) (26) in addition to the Hamilton Depression Scale (HAM-D) and Hamilton Anxiety Inventory (HAM-A) to quantify mood symptoms. The CSSRS assesses a range of SB, including SI (wishing to be dead and suicidal thoughts with and without methods, plan, and intent) and SA (history of actual, interrupted (interrupted by another being/situation), and aborted (self-interrupted) suicide attempts). Higher scores on the CSSRS indicate a higher level of SB (e.g., 1 is a lifetime history of wishing to be dead, 9 is a lifetime history of suicide attempt). Participants were included in the SI group if they reported wishing to be dead or suicidal thoughts with or without methods, plan and intent. Those who reported actual, aborted, or interrupted attempts were placed in the SA group. The HAM-D (27) and HAM-A (28) are clinician-administered mood assessments that include items on depressed mood, feelings of guilt, insomnia, agitation, anxious mood, fears, and physiological symptoms such as heart palpitations and choking sensation. On the HAM-D, a total score of 17 or greater is considered moderate up to severe depression; on the HAM-A a score of 18 or greater is considered moderate up to severe anxiety (28).

### 2.4 MRI acquisition and analysis

A subsample of 77 Veteran participants completed MRI data acquisition. MRI brain data were obtained using a 3.0 Tesla Siemens Magnetom Verio scanner and a standard 12-channel head coil. Using a T1-weighted 3D MPRAGE GRAPPA sequence, the axial plane T1-weighted images were acquired using the following parameters: Echo Time (TE)=3.42 ms, Repetition Time (TR)=2000 ms, Inversion Time (TI)=1100 ms, Flip Angle=8°, 256×256 acquisition matrix, 160 slices, and 1.0 mm slice thickness.

Image data were transferred from the scanner in DICOM format and coded. Intracranial volume (ICV) and ACC volume were analyzed using Freesurfer version 5.3 image analysis suite (http://surfer.nmr.mgh.harvard.edu/) and regions of interest were parcellated based on the Desikan-Killiany atlas. Both ICV and ACC volume measures were included in the statistical outputs that are automatically generated via the recon-all script (FreeSurfer processing stream). Description of segmented volume measurements and total ICV determination using an atlas-based normalization procedure using the MNI305 space are detailed in prior work (29–31). All processed images were visually inspected for quality control to ensure that the gray matter (GM) and white matter (WM) boundaries were accurately defined. Additionally, all Veteran MRI data were securely reviewed by a board-certified neuroradiologist to screen for gross pathology.

### 2.5 Statistical analysis

Demographic characteristics were compared between the groups using the *t*-test and Chi-square test for the continuous and categorical variables, respectively. Between-group differences in clinical outcome measures, such as the Ruff 2 & 7, HAM-A, and HAM-D scores, were analyzed using linear regression models with age and sex included as covariates. A Bonferroni correction was applied to account for the number of outcomes examined (0.05/6 = 0.008). In the subsample with structural imaging data, the group differences in ICV were tested using linear regression models. Correlations between the Ruff 2 & 7 scores and ACC volumetric measures, which were adjusted for total ICV, were examined using a linear regression model. All analyses included age and sex as covariates. A two-tailed p < 0.05 was considered statistically significant for the regression analysis. All analyses were performed using Stata version 18.0 (StataCorp, College Station, TX, USA).

## 3 Results

### 3.1 Demographic data, clinical measures, and attention scores

Participant average age was 38.39 years old. Forty Veterans reported no history of SB (11 females, 29 males), including ideation and attempts. Fifty-five Veterans reported a history of SI (19 females, 36 males) and thirty-four reported a history of SA (8 females, 26 males). Veteran groups did not differ on important demographic features, including education and handedness. (See **Table 1**.) Across the sample, 16 participants reported HAM-A scores of 18 or greater (range = 18-35) and 16 reported HAM-D scores of 17 or greater (range = 17-28). Ten individuals reported a HAM-A score of 18+ and a HAM-D score of 17+, suggesting comorbid depression and anxiety. Additionally, T-scores generated from Ruff 2 & 7 performance measures showed that on the ADS subscale 19 participants performed below average (T = 19-39) and 22 performed above average (T = 61-78). On the Ruff 2 & 7 CSS, 19 participants performed below average (T = 19-39) and 19 performed above average (T = 61-77). On the Ruff 2 & 7 Speed score, 15 participants scored below average (T = 19-39) and 25 participants scored above average (T = 61-77), while on the Ruff 2 & 7 Accuracy score, 16 participants scored below average (T = 21-39) and 1 participant scored above average (T = 61). Two male participants in the HC group and one male participant in the SA group completed clinical measures of SB, mood, and anxiety. However, as they did not have completed Ruff 2 & 7 raw data, they were removed from the analyses.

**Table 1:** Veteran group differences on demographics and measures of depressive and anxious symptoms, and Ruff 2 & 7 attention scores.

	HC (N = 40)	SI (N = 55)	SA (N = 34)	*p-*value	
				HC vs. SA	SI vs. SA
Age, years	37.17 (9.47)	37.37 (8.74)	40.64 (9.03)	0.10	0.10
Sex					
Female, N (%)	11 (27.50)	19 (34.55)	8 (23.53)	0.70	0.27
Male, N (%)	29 (72.50)	36 (65.45)	26 (76.47)		
Education, years	14.75 (1.90)	15.25 (2.23)	14.81 (2.07)	0.90	0.34
Handedness					
Left, N (%)	6 (15.79)	3 (5.77)	2 (5.88)	0.18	0.98
Right, N (%)	32 (84.21)	49 (94.23)	32 (94.12)		
HAM-A	4.05 (4.40)	9.27 (7.00)	14.91 (9.14)	< 0.001***	0.003*
HAM-D	2.65 (3.28)	7.42 (5.92)	13.44 (7.01)	< 0.001***	< 0.001***
ADS Raw	158.97 (27.67)	159.71 (30.21)	140.15 (29.20)	0.02	0.007*
ADS T	52.47 (9.68)	52.27 (11.24)	46.64 (10.10)	0.02	0.02
CSS Raw	136.63 (24.14)	138.75 (24.42)	123.00 (21.98)	0.02	0.003*
CSS T	50.84 (11.24)	50.57 (11.10)	45.97 (10.19)	0.047	0.03
Speed T	53.63 (10.19)	52.91 (10.94)	47.91 (10.18)	0.02	0.03
Accuracy T	49.11 (9.36)	48.73 (8.76)	48.34 (8.57)	0.69	0.86

Data are shown as mean (standard deviation) unless otherwise specified. *Bonferroni-corrected *p* < 0.05; ***Bonferroni-corrected *p* < 0.001. Age and sex were included as covariates in the group comparison of clinical outcome variables. HC, no SB; SI, suicidal ideation; SA, suicide attempt; HAM-A, Hamilton Anxiety Inventory; HAM-D, Hamilton Depression Scale; ADS, Ruff 2 & 7 Automatic Detection Speed; CSS, Ruff 2 & 7 Controlled Search Speed. Ruff 2 & 7 data missing for three males (1 SA and 2 HC).

### 3.2 Attention and mood differences by suicide behavior group

**Figure 1:**
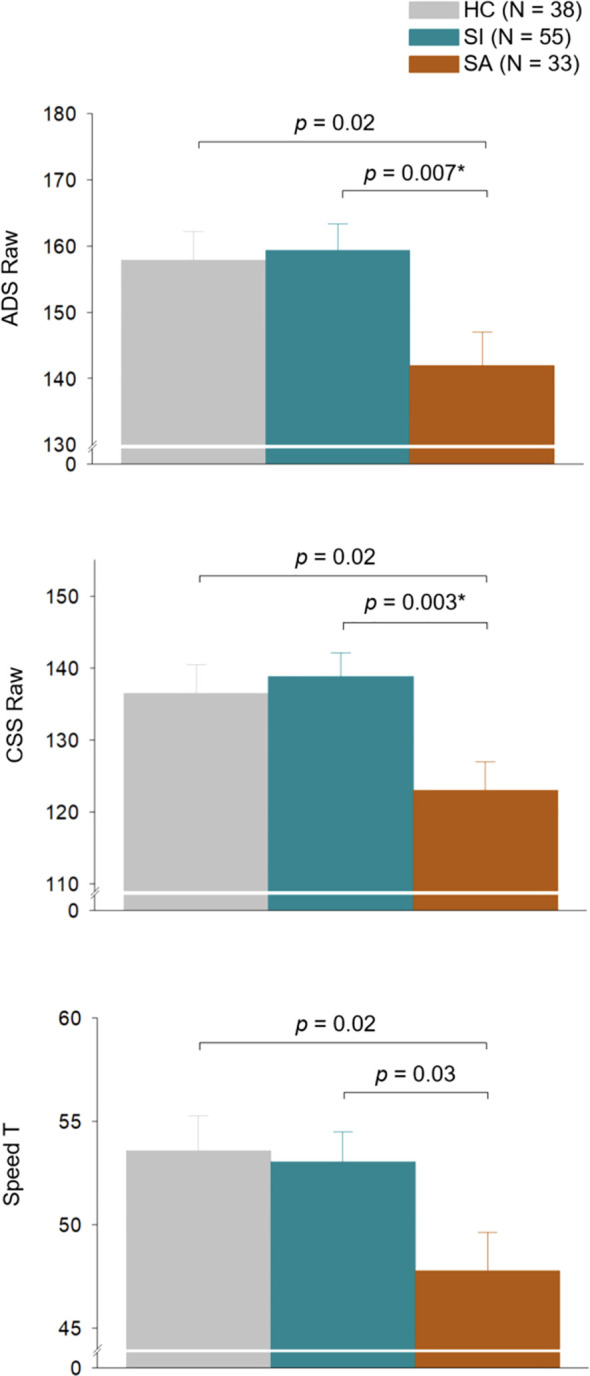
Attentional performance on Ruff 2 & 7 Automatic Detection Speed, Controlled Search Speed, and Total Speed of HC, SI, and SA groups. Each bar represents the predicted mean of each group adjusted for age and sex, and the error bar represents the standard error of the mean. Two participants in the HC group and one participant in the SA group did not have Ruff 2 & 7 data and were therefore excluded from the analysis. *Bonferroni-corrected *p*< 0.05. HC, no suicide behavior; SI, suicidal ideation; SA, suicide attempt; ADS, Ruff 2 & 7 Automatic Detection Speed; CSS, Ruff 2 & 7 Controlled Search Speed.

Veterans with a history of SA performed more slowly on Ruff ADS and CSS compared to Veterans with a history of SI and those with no SB (**Figure 1** and **Table 1**). After Bonferroni correction for multiple variables, SI and SA groups differed on ADS and CSS. There was no significant difference between HC and SI on ADS or CSS. SA also performed more poorly on Ruff 2 & 7 Total Speed compared to SI and HC, although this did not survive Bonferroni correction. There was no significant difference between HC and SI. Groups did not differ on Total Accuracy (See **Table 1**).

HC, SI, and SA showed significant differences on HAM-A and HAM-D scores with SA evidencing the highest HAM-A and HAM-D scores, followed by those with SI and then HC. Significant differences on Ruff 2 & 7 ADS, CSS, and Total Speed scores remained after controlling for both depressive and anxious symptoms (ADS Raw: HC vs SA *p =* 0.07, SI vs SA *p =* 0.02; ADS T-scores: HC vs SA *p =* 0.04; SI vs SA *p =* 0.02; CSS Raw: HC vs SA *p =* 0.07, SI vs SA *p =* 0.006; CSS T-scores: HC vs SA *p =* 0.08; SI vs SA *p =* 0.03; Total Speed T-scores: HC vs SA *p =* 0.047, SI vs SA *p =* 0.03 after controlling for combined HAM-A and HAM-D scores). However, after Bonferroni correction, only CSS remained significant (*p* = 0.006; **Table 1** and **Supplementary Table S1**).

### 3.3 Sex differences in attention by suicide behavior group

**Figure 2:**
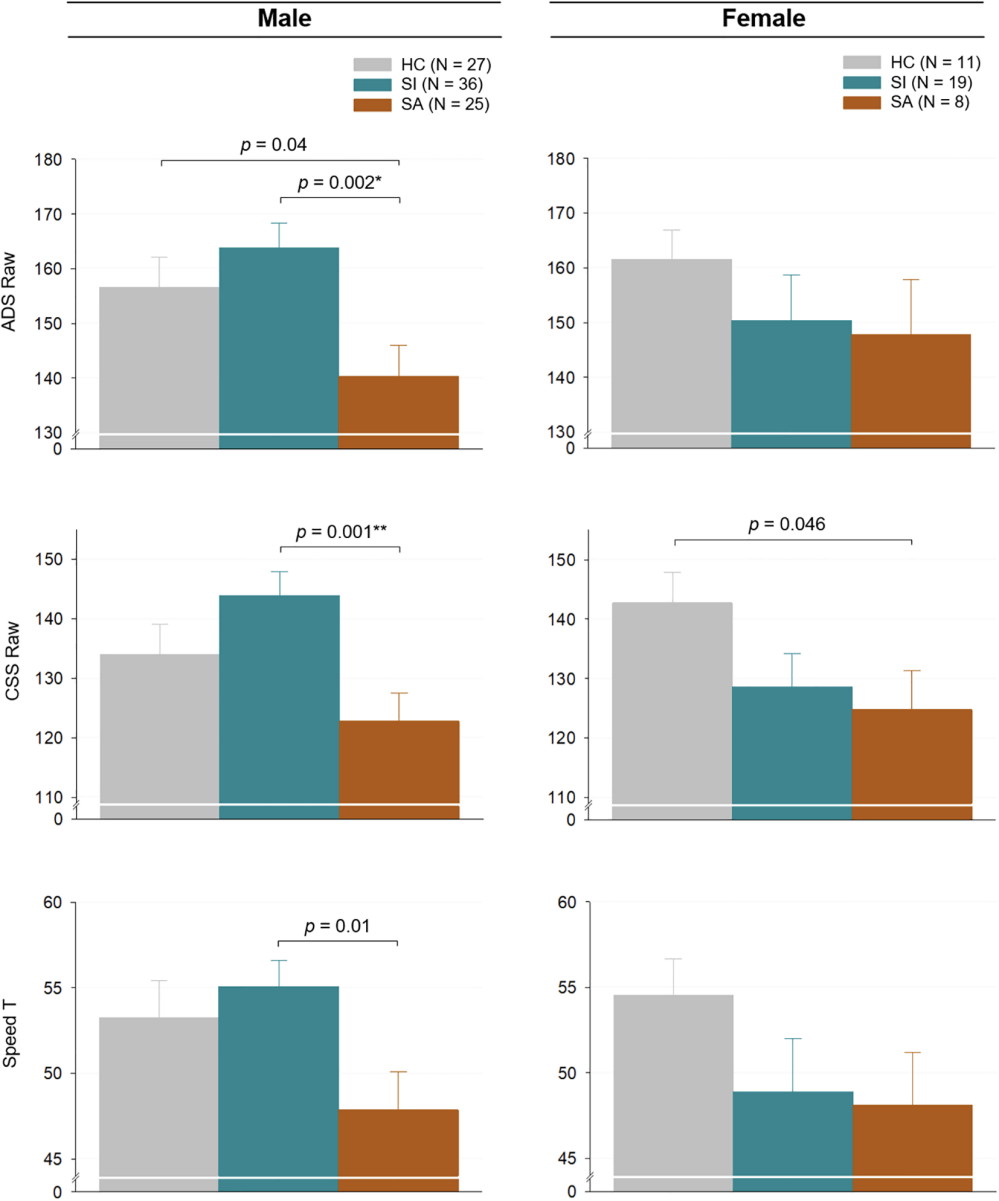
Attentional performance on the Ruff 2 & 7 Automatic Detection Speed, Controlled Search Speed, and Total Speed by SB and sex. Each bar represents the predicted mean of each group adjusted for age and sex, and the error bar represents the standard error of the mean. Two participants in the male HC group and one participant in the male SA group did not have Ruff 2 & 7 data and were therefore excluded from the analysis. *Bonferroni-corrected *p* < 0.05; **Bonferroni-corrected *p* <0.01; SB, suicidal behavior; HC, no suicide behavior; SI, suicidal ideation; SA, suicide attempt; ADS, Ruff 2 & 7 Automatic Detection Speed; CSS, Ruff 2 & 7 Controlled Search Speed.

Analyses also were run to examine the impact of sex on attention differences in the HC, SA, and SI groups. (See **Figure 2** and **Supplementary Table S2**.) In these analyses, males with SA performed significantly slower on ADS compared to HC and SI. This result remained significant when controlling for HAM-A and HAM-D (ADS Raw: HC vs SA *p =* 0.02; SI vs SA *p* < 0.001; ADS T-scores: HC vs SA *p =* 0.02; SI vs SA *p =* 0.002). Differences on ADS speed performance between males with SA and males with SI also survived Bonferroni correction for multiple variables, although differences between HC males and males with SA did not. Females showed no significant between-group differences on ADS. Males with SA performed more slowly on the CSS compared to males with SI, which remained significant after controlling for HAM-A and HAM-D and Bonferroni correction (CSS Raw: SI vs SA *p* < 0.001). Females with SA performed more slowly than female HC on the CSS. However, this finding did not remain significant after controlling for HAM-A and HAM-D in females. Males with SA also performed more slowly than males with SI on the Total Speed score, which remained significant after controlling for HAM-A and HAM-D and Bonferroni correction (Total Speed T-score: SI vs SA *p =* 0.004). There were no significant between group difference for females on Total Speed.

### 3.4 Anterior cingulate cortex volume and attentional performance

Seventy-seven Veterans ages 18-55 also completed MRI on a 3T Siemens scanner. In this sample, twenty-five Veterans reported no history of SB (5 females, 20 males), including ideation and attempts. Thirty Veterans reported a history of SI (6 females, 24 males) and 22 reported a history of SA (3 females, 19 males). Importantly, there were no significant differences in age, sex, HAM-A, HAM-D, SB, or attention scores between Veteran group who completed MRI and those who did not.

**Figure 3:**
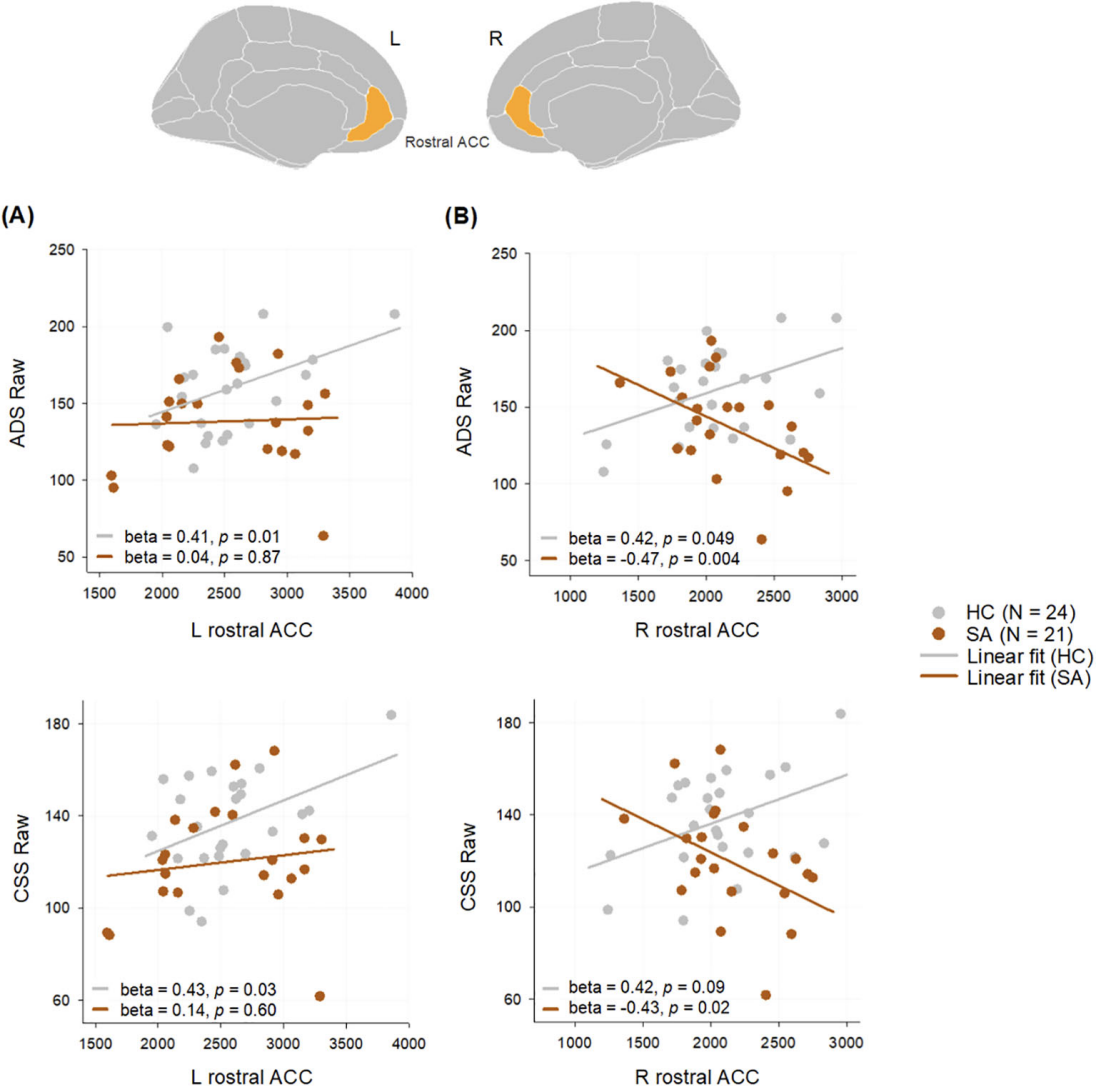
Correlations between **(A)** left and **(B)** right rostral ACC volumes and ADS and CSS scores in SA and HC groups. Age and sex were included as covariates in correlations. The volumetric measures adjusted for total intracranial volume were used as outcomes variables. One participant in each group did not have Ruff 2 & 7 data and was therefore excluded from correlations. ACC, anterior cingulate cortex; ADS, Ruff 2 & 7 Automatic Detection Speed; CSS, Ruff 2 & 7 Controlled Search Speed; SA, suicide attempt; HC, no suicide behavior; L, left; R, right.

Due to small sample size, we were unable to complete imaging analyses by sex but instead focused on SB. Individuals with SA showed significantly reduced ICV compared to HC in both sexes (total sample: HC vs. SI, P = 0.19; HC vs. SA, P = 0.003; SI vs. SA, P = 0.10). Left rostral ACC GM volume correlated positively with ADS and CSS scores in HC but not SA. Right rostral ACC GM volume negatively correlated with ADS and CSS scores in the SA group. Right rostral ACC GM volume was also positively correlated with ADS raw in the HC group. (See **Figure 3**.) Right rostral ACC WM volume also correlated positively with ADS raw and CSS raw only in HC (**Table 2**).

**Table 2:** Correlations between gray and white matter volume in the rostral ACC and Ruff 2 & 7 scores in HC and SA.

	HC (N = 24)			SA (N = 21)		
	Beta	*t*	*p*	Beta	*t*	*p*
Gray matter						
R rostral ACC						
ADS Raw	0.42	2.10	0.049*	-0.47	-3.36	0.004**
CSS Raw	0.42	1.79	0.09	-0.43	-2.70	0.02*
L rostral ACC						
ADS Raw	0.41	2.67	0.01*	0.04	0.17	0.87
CSS Raw	0.43	2.27	0.03*	0.14	0.53	0.60
White matter						
R rostral ACC						
ADS Raw	0.52	2.96	0.008**	0.16	0.80	0.43
CSS Raw	0.61	2.74	0.01*	0.29	1.48	0.16
L rostral ACC						
ADS Raw	0.21	1.34	0.20	0.07	0.39	0.70
CSS Raw	0.21	1.03	0.31	0.17	0.83	0.42

Age and sex were included as covariates in all analyses. * *p* < 0.05; ** *p* < 0.01. The volumetric measures adjusted for total intracranial volume were used as outcomes variables. One participant in each group did not have Ruff 2 & 7 data and was therefore excluded from correlations.

## 4 Discussion

Veterans with a history of suicide attempts showed significant decreases in ADS and CSS compared to those without suicide attempts. Poor sustained and selective attention was not fully accounted for by symptoms of depression or anxiety, suggesting attentional differences beyond acute mood. These findings support previous reports of cognitive deficits, particularly changes in attention associated with SB and extend the results to the Veteran population (13).

At the time of assessment Veterans in all three SB groups reported mean anxiety and depression scores below clinical cutoffs. Even individuals with a history of SA endorsed average mean depression and anxiety scores in the mild to moderate range. Nonetheless, when associations between cingulate morphometry and cognitive performance were examined, they showed decreases in attentional speed that were related to ACC volume. Moreover, while attention speed was statistically different in SA, the mean scores for all three groups fell within the average performance range. This highlights the possibility that even when individuals with a history of SA score in the average range on attention and mood measure scores, differences in underlying neurobiological correlates may remain.

One concern in interpreting data from studies of neurocognition and SB has been the potential confounding effects of depression and anxiety on neurocognitive performance, as significant research has shown psychomotor retardation and decreased attention in individuals with depression and anxiety (32). In the current study differences in attention performance by SB group did not change when depressive and anxious symptoms were included in the model for males. In contrast, the same associations became non-significant when controlling for HAM-A and HAM-D in females. Previous studies have shown consistency in Ruff 2 & 7 performance irrespective of mood symptoms; however, these earlier analyses did not complete analyses by sex (33, 34). It is therefore unclear whether depressive and anxious symptoms affect attention performance more in females compared to males, as our sample may suggest. Future studies should include sex as a variable in analyses. In addition, other research examining ACC and medial prefrontal cortex (mPFC) activation has found changes in some individuals with depression but not others. Yang and colleagues divided patients with depression into groups determined by a premorbid intelligence quotient compared to current estimated IQ. The two groups, identified as deteriorated IQ (DIQ) and preserved IQ (PIQ) were shown to differ on multiple cognitive domains, as the DIQ group showed impaired motor speed and decreased cognitive flexibility in addition to increased activation of the ACC and mPFC. The PIQ and HC groups did not show these changes. The authors suggest that it may be useful to classify cognitive changes associated with depression, as not all individuals with depression evidence heterogeneous changes in neurocognitive performance and brain activation depending on a host of premorbid factors (35).

The current study results on Ruff attention speed and ACC volume support prior research findings demonstrating an association between the ACC and attentional functions. There has been limited research describing the role of the ACC in execution of motor tasks in healthy controls screened for Axis I diagnosis (36, 37). The current study offers pilot support for the role of the ACC in motor tasks as well, as ACC volume was related to Ruff 2 & 7 speed but not accuracy. If ACC volume were related to all aspects of attention, it would be expected that performance on accuracy would also have differed between groups. Importantly, the current results emphasize the importance of the rostral ACC, which has been connected to cognitive control (38).

Differences in the association between attention performance speed and ACC volume of those with SA and HC also highlight the importance of examining different types of SB, as many clinical and neurobiological differences between individuals with differing types of SB have not been examined. While some theories suggest that SI transitioning to SA is a progression with an ideation to action framework (39), factors that may underly the transition from SI to SA remain unclear. It has been reported that only 29% of people with a lifetime history of suicidal ideation will go on to make an attempt (40). Given this, it is critical to understand what leads some to make this potentially lethal progression, whereas others do not. Many established risk factors for suicidality, such as depression and impulsivity, predict the presence of suicidal thoughts but do not explain the transition from ideating to attempting (39, 41). Interpreting the differences between the groups is made more difficult by studies historically not defining ideators and attempters as distinct groups (42).

The current study also highlights the importance of rostral ACC in relation to attention in individuals with SB versus HC. These findings augment prior research using diffusion tensor imaging to identify WM changes in individuals with SB, particularly in areas responsible for executive function, decision making, and emotion processing, such as the prefrontal cortex, orbitofrontal cortex, corpus collosum, and default mode networks (43). The current findings and others support the importance of including multimodal neuroimaging techniques to examine not only regional volumes but also microstructural alterations in WM.

Results from the current study are in contrast to several prior studies showing no differences in attention between individuals with a history of SB and those without. However, a number of previous reports showing no association between attention and SB focused on individuals with specific psychiatric diagnoses, including bipolar disorder (44, 45) and schizophrenia (46). The current sample included Veterans with a variety of psychiatric diagnoses. Importantly, attentional differences remained even after controlling for depressive and anxious symptoms, suggesting that mood was not driving the attentional results on the day of assessment.

Findings showing attention deficits in individuals with SA also have important treatment implications (6). Many current psychological interventions for suicide risk and prevention rely on therapist and patient discussing symptoms, triggers, coping strategies, or alternative behaviors to suicide. These interventions may require a patient to sustain attention across many minutes in order to identify thoughts, behaviors, and alternatives. Moreover, Veterans with SA may take longer to process and respond to information compared to others. Common talk therapy strategies may be less effective if the Veteran cannot attend, encode, process information quickly, and remember to utilize the information learned in therapy. This challenge extends to other cognitive processes as well, as individuals with SB may have decreases in working memory, cognitive control, executive function, or other cognition (3). Future research should continue to explore the range of cognitive processes in individuals with SB. Importantly, strategies such as having patients write information down so that they may revisit it in written form (e.g., written safety plan) or checking in regularly to ensure the patient is able to maintain attention during the therapy work may be helpful. Other cognitive rehabilitation interventions focusing on restorative and/or compensatory treatment such as paper and pencil or computerized cognitive training approaches might also improve cognitive processes. Future research could benefit from examining these interventions particularly in participants with SI compared to SA. Deficits in selective attention may make Veterans with SB more likely to focus on feelings of hopelessness rather than being able to readily shift focus to new skills or to changing cognitions. Interventions such as Dialectical Behavior Therapy, which focuses on distraction and redirecting attention to other skills may be of use in populations with SB (6).

The current study has several limitations. First, all data are cross-sectional, limiting the ability to determine whether differences in attention and associations to ACC volume were present prior to SB or the result of SB or comorbid symptoms. This is particularly important in light of debate around whether SB is a state or trait phenomenon (39). Our data support a trait-approach, as we found that differences between SI, SA, and HC endured long after the SB had resolved. However, longitudinal data would address this debate in further detail and inform knowledge of changes in SB, attention, and ACC volume over time. In addition, female Veterans are underrepresented in this sample. Although we focused on recruiting a larger number of females than are often included in studies with Veterans, the majority of the participants in this study still were male. This is a common limitation in Veteran literature and studies should endeavor to include more females to examine not only differences in SB but also to include sex as an independent variable. Lastly, Veterans in this study were recruited with many inclusion criteria and few exclusion criteria. This approach ensures greater generalizability but less specificity in any singular disorder or comorbidities.

In summary, the present study showed significant sustained and selective attention differences between female and male Veterans with SI, SA, and HC, particularly in speed. Groups also differed on associations between ACC volume and attention, suggesting that SB is related to attention and cingulate brain volume differently in SI, SA, and HC. Future research should continue to examine different types of SB in relation to neurocognition and neurobiological markers by sex with the goal of improving both assessment and treatment.

## Data Availability

The raw data supporting the conclusions of this article will be made available by the authors, without undue reservation.
