# Supplementary material for: Attention speed and anterior cingulate cortex volume in female and male veterans with suicide ideation and attempts
Source: Front Psychiatry. 2025 Jan 22;15:1495046. doi: 10.3389/fpsyt.2024.1495046 (PMC11795208; doi:10.3389/fpsyt.2024.1495046)
Supplement: Supplementary file 1 [file SupplementaryFile1.docx]

**Supplementary Table 1**. Veteran group differences on Ruff 2 & 7 attention scores after adjusting for HAM-A and HAM-D

|  | HC  (N = 38) | SI  (N = 55) | SA  (N = 33) | *p-*value | |
| --- | --- | --- | --- | --- | --- |
|  |  |  |  | HC vs. SA | SI vs. SA |
| ADS Raw | 158.97 (27.67) | 159.71 (30.21) | 140.15 (29.20) | 0.07 | 0.02 |
| ADS T | 52.47 (9.68) | 52.27 (11.24) | 46.64 (10.10) | 0.04 | 0.02 |
| CSS Raw | 136.63 (24.14) | 138.75 (24.42) | 123.00 (21.98) | 0.07 | 0.006* |
| CSS T | 50.84 (11.24) | 50.57 (11.10) | 45.97 (10.19) | 0.08 | 0.03 |
| Speed T | 53.63 (10.19) | 52.91 (10.94) | 47.91 (10.18) | 0.047 | 0.03 |
| Accuracy T | 49.11 (9.36) | 48.73 (8.76) | 48.34 (8.57) | 0.64 | 0.84 |

*Bonferroni corrected *p* < 0.05 after adjusting for HAM-A and HAM-D.

**Supplementary Table 2**. Female and male Veteran group differences on Ruff 2 & 7 attention scores after adjusting for HAM-A and HAM-D

|  | **Male (N = 88^a^)** | | **Female (N = 38)** | |
| --- | --- | --- | --- | --- |
|  | *p-*value | | *p-*value | |
|  | HC vs. SA | SI vs. SA | HC vs. SA | SI vs. SA |
| ADS Raw | 0.02 | <0.001*** | 0.71 | 0.84 |
| ADS T | 0.02 | 0.002* | 0.54 | 0.96 |
| CSS Raw | 0.10 | <0.001** | 0.25 | 0.95 |
| CSS T | 0.19 | 0.01 | 0.16 | 0.85 |
| Speed T | 0.07 | 0.004* | 0.28 | 0.98 |
| Accuracy T | 0.75 | 0.93 | 0.46 | 0.51 |
| ^a^ Ruff 2 & 7 data missing for three males (2 HC and 1 SA); *Bonferroni corrected *p* < 0.05; **Bonferroni corrected *p* < 0.01; ***Bonferroni corrected *p* < 0.001 after adjusting for HAM-A and HAM-D. | | | | |
